# Clinical manifestations, progression and treatment of post-traumatic hydrocephalus accompanied by contralateral subdural effusion after decompressive craniectomy: a single-center retrospective study

**DOI:** 10.3389/fneur.2026.1833064

**Published:** 2026-05-08

**Authors:** Jiaji Qiu, Yu Yan, Weiming Liu, Guoyi Gao

**Affiliations:** 1 Department of Neurosurgery, Beijing Tiantan Hospital, Capital Medical University, Beijing, China; 2 Beijing Key Laboratory of Central Nervous System Injury, Beijing Neurosurgical Institute, Capital Medical University, Beijing, China

**Keywords:** contralateral subdural effusion, cranioplasty, decompressive craniectomy, post-traumatic hydrocephalus, subdural-peritoneal shunt, traumatic brain injury, ventriculoperitoneal shunt

## Abstract

**Background:**

Post-traumatic hydrocephalus (PTH) and contralateral subdural effusion (cSDE) are common complications after decompressive craniectomy (DC) in severe traumatic brain injury. However, their concurrent occurrence poses diagnostic and therapeutic challenges. This study aims to analyze the clinical features, radiological progression, and treatment outcomes of this condition.

**Methods:**

We retrospectively reviewed 436 patients with severe traumatic brain injury who underwent DC at Beijing Tiantan Hospital between September 2018 and January 2026. From this cohort, 21 patients who developed both PTH and cSDE were selected. Their clinical data, radiological features, treatment modalities, and outcomes were analyzed. PTH was defined as Evans’ index > 0.3 with clinical symptoms; cSDE was defined as a hypodense crescentic fluid collection over the hemisphere contralateral to the craniectomy site.

**Results:**

The overall incidence of concurrent post-traumatic hydrocephalus and contralateral subdural effusion was 4.8% (21/436) among patients who underwent DC, with cSDE occurring at a mean of 15.6 days post-craniectomy. Among the 21 patients, 12 presented with neurological deterioration and underwent burr-hole drainage, and 2 underwent Ommaya reservoir placement due to neurological deterioration. Six patients underwent cranioplasty alone, and 4 patients received cranioplasty combined with ventriculoperitoneal shunt. Seven patients underwent cranioplasty and subsequently received ventriculoperitoneal shunt. Two patients were treated with subdural-peritoneal shunt for refractory subdural effusion, including one patient who, due to the severity of both hydrocephalus and subdural effusion, underwent ventriculoperitoneal shunt and subdural-peritoneal shunt followed by elective cranioplasty.

**Conclusion:**

PTH with concomitant cSDE requires individualized, staged management. Cranioplasty may resolve cSDE but often unmasks underlying hydrocephalus, necessitating close postoperative monitoring and timely shunt placement.

## 1 Introduction

Decompressive craniectomy (DC) is a life-saving intervention for patients with severe traumatic brain injury (TBI) complicated by refractory intracranial hypertension (1). Although DC effectively reduces mortality, it is associated with a spectrum of complications, notably post-traumatic hydrocephalus (PTH) and subdural effusion (SDE) (2–4).

Contralateral subdural effusion (cSDE) refers to fluid accumulation over the cerebral hemisphere opposite the craniectomy site, with reported incidences ranging from 4.4 to 7.3% (2–5). While some cSDEs remain asymptomatic and resolve spontaneously, others may exert mass effect and precipitate neurological deterioration, necessitating active intervention (2, 3). Various treatment options have been proposed, including conservative management, burr-hole drainage, Ommaya reservoir implantation, subduroperitoneal shunting, and cranioplasty (6–9).

Post-traumatic hydrocephalus represents another frequent complication following DC, with incidence rates varying considerably across studies (1, 4). Its pathogenesis involves disruption of cerebrospinal fluid (CSF) dynamics, impaired absorption at the arachnoid granulations, and altered intracranial pressure (ICP) waveforms consequent to the cranial defect (1, 4, 10).

Notably, several studies have documented a high rate of subsequent hydrocephalus among patients who develop cSDE after DC. Su et al. (2) reported that 46.2% of patients with cSDE eventually required ventriculoperitoneal (VP) shunt placement for hydrocephalus. Similarly, Wang et al. (4) described a patient whose cSDE resolved after cranioplasty but later developed symptomatic hydrocephalus necessitating VP shunt. These observations suggest a pathophysiological link between cSDE and PTH, possibly attributable to shared disturbances in CSF dynamics (4, 11, 12).

When PTH and cSDE coexist, clinical management becomes particularly challenging. The presence of cSDE may mask or mimic the symptoms of hydrocephalus (2, 4), and the optimal treatment sequence—whether to prioritize cranioplasty, burr-hole drainage, VP shunt placement, or combined procedures—remains a subject of debate (3, 7, 8, 11).

The mechanisms underlying the concurrent development of PTH and cSDE are not fully understood. Several theories have been proposed. The “arachnoid valve” theory posits that traumatic tearing of the arachnoid membrane creates a one-way valve, permitting CSF to flow into the subdural space (4, 5, 11). The “pressure gradient” theory suggests that rapid intraoperative brain shift following dural opening generates a pressure differential between the hemispheres, thereby enlarging the contralateral subdural space (4, 5, 12). The “CSF absorption” theory implicates impaired function of the arachnoid granulations due to a flattened dicrotic ICP waveform after craniectomy, leading to reduced CSF absorption and subsequent fluid accumulation (1, 4, 10).

In recent years, cranioplasty has emerged as an effective treatment for refractory cSDE (3, 4, 7, 10, 11, 13). By restoring the “closed box” of the cranium, cranioplasty normalizes ICP dynamics and may resolve both cSDE and hydrocephalus (4, 11). However, as emphasized by Wang et al. (4) and Salunke et al. (11), cranioplasty alone may be insufficient when hydrocephalus is already established, and some patients subsequently require VP shunt placement.

Despite increasing recognition of this clinical entity, studies specifically addressing the coexistence of PTH and cSDE remain scarce. The existing literature is largely confined to case reports (4, 6, 9, 10, 13–15) or small case series (2–4, 7, 11), and optimal management protocols have yet to be established. Therefore, this study aimed to: (1) characterize the clinical and radiological features of PTH with concomitant cSDE after DC; (2) evaluate the outcomes of various treatment strategies; and (3) propose a preliminary management algorithm based on our single-center experience.

## 2 Materials and methods

### 2.1 Study design and setting

This study is a single-center, retrospective cohort study that included medical data from patients treated at Beijing Tiantan Hospital between September 2018 and January 2026. All patients enrolled in this study had signed informed consent forms for the use of their clinical diagnostic and treatment data upon admission. Due to its retrospective nature, the requirement for informed consent specific to this study was waived.

### 2.2 Patient selection

**Figure 1:**
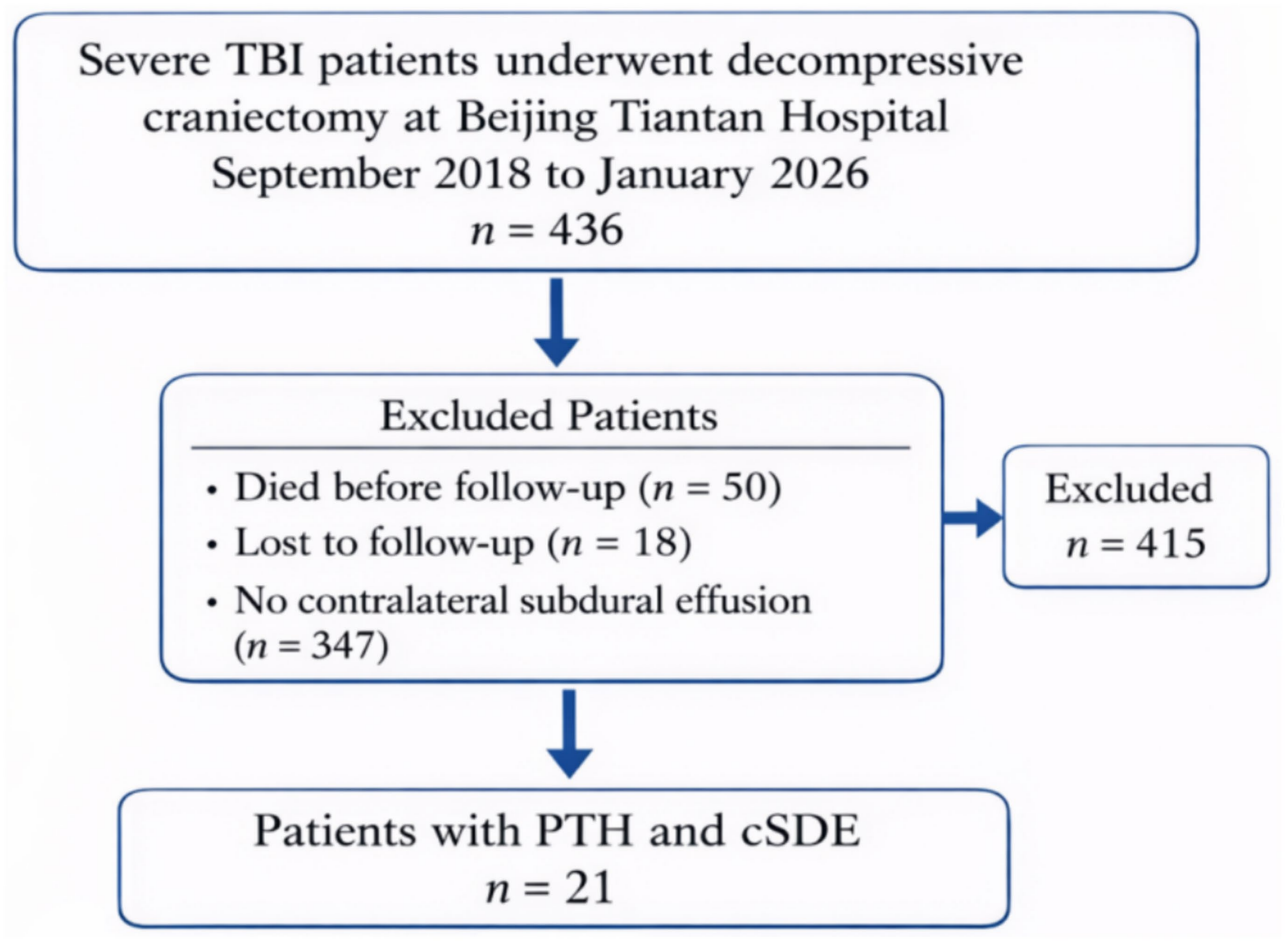
Flow diagram of patient selection and study cohort.

We reviewed the medical records and imaging data of all patients with severe TBI who underwent DC at our hospital between September 2018 and January 2026. Patient selection and study cohort flow diagram is shown in Figure 1. The baseline characteristics of the patients with PTH and cSDE included in this study are shown in Table 1.

**Table 1:** Baseline characteristics of patients with PTH and cSDE.

Characteristic	Value (*N* = 21)
Age (years), mean ± SD	55.3 ± 14.7
Male sex, *n* (%)	16 (76.2%)
Mechanism of injury, *n* (%)	
Motor vehicle accident	9 (42.9%)
Fall	7 (33.3%)
Other	5 (23.8%)
Admission GCS, median (IQR)	8 (5–11)
Side of craniectomy, *n* (%)	
Right	15 (71.4%)
Left	6 (28.6%)
Timing of DC from injury (hours), mean ± SD	6.8 ± 4.2
Concurrent hematoma evacuation, *n* (%)	18 (85.7%)

Patients were included if they met the following criteria: age ≥ 18 years, underwent unilateral DC for severe TBI, developed both PTH and cSDE during follow-up, and had a minimum follow-up period of 6 months. Patients were excluded if they had pre-existing hydrocephalus or a history of prior shunt placement, died within 30 days post-DC due to causes unrelated to the study conditions, had incomplete medical records or imaging data, or were lost to follow-up.

### 2.3 Definitions

Decompressive craniectomy was defined as the surgical removal of a bone flap with a minimum diameter of 12 cm for intracranial pressure control (1); cSDE was defined as a hypodense crescentic fluid collection over the cerebral convexity contralateral to the craniectomy site diagnosed on CT scan (2, 3); PTH was defined as an Evans’ index (the ratio of the maximum width of the frontal horns to the maximum internal diameter of the skull) greater than 0.3 accompanied by clinical symptoms such as impaired consciousness, psychomotor retardation, gait disturbance, or urinary incontinence (2, 4); and symptomatic cSDE was defined as cSDE causing midline shift >5 mm or neurological deterioration (e.g., a decrease in GCS score of ≥2 points or new focal deficits) attributable to the effusion.

### 2.4 Data collection

Demographic, clinical, radiological, and outcome data were collected from electronic medical records, including demographics such as age, sex, mechanism of injury, and admission Glasgow Coma Scale (GCS) score; surgical data including indication for DC, side of craniectomy, timing of DC from injury, and concurrent procedures (e.g., hematoma evacuation); clinical manifestations at cSDE diagnosis such as headache, decreased consciousness, bulging flap, seizures, and focal deficits, along with the time from DC to symptom onset, time from DC to cSDE diagnosis, and time from cSDE to PTH diagnosis; radiological features assessed on serial CT scans including cSDE thickness (maximal width in mm), presence and degree of midline shift (mm), Evans’ index at each time point, ventricular configuration, and presence of brain herniation through the cranial defect; treatment modalities categorized according to initial treatment approach as conservative management (observation, head-down positioning, fluid management), burr-hole drainage (with or without Ommaya reservoir placement), cranioplasty alone, VP shunt alone, staged cranioplasty followed by VP shunt, or simultaneous cranioplasty and VP shunt; and outcome measures including resolution of cSDE (complete or partial on follow-up CT), need for subsequent surgical interventions, Glasgow Outcome Scale (GOS) score at 6 months (favorable: GOS 4–5; unfavorable: GOS 1–3), and complication rates (infection, hemorrhage, shunt malfunction).

### 2.5 Statistical analysis

Statistical analysis was performed using IBM SPSS Statistics 27.0.1. Continuous variables were expressed as mean ± standard deviation or median (interquartile range) as appropriate. Categorical variables were expressed as frequencies and percentages.

For comparisons between groups, continuous variables were analyzed using Student’s t-test or Mann–Whitney U test, and categorical variables were analyzed using the chi-square test or Fisher’s exact test, as appropriate. Univariate and multivariate logistic regression analyses were performed to identify factors associated with poor outcome (GOS 1–3), with variables showing *p* < 0.01 in univariate analysis entered into the multivariate model. Statistical significance was set at *p* < 0.05.

## 3 Results

### 3.1 Clinical manifestations

The clinical characteristics and time course of cSDE and PTH development are presented in Table 2. The results show that the mean interval from DC to cSDE diagnosis was 15.6 days (range: 5–28 days). At the time of cSDE diagnosis, 5 patients (23.8%) were asymptomatic, with the effusion detected on routine follow-up CT, while 16 patients (76.2%) presented with clinical deterioration.

**Table 2:** Clinical characteristics and timeline.

Parameter	Value
Time from DC to cSDE diagnosis (days), mean (SD)	15.6 (6.1)
Time from DC to cSDE diagnosis (days), range	5 ~ 28
Asymptomatic at cSDE diagnosis, *n* (%)	5 (23.8%)
Symptomatic at cSDE diagnosis, *n* (%)	16 (76.2%)
Time from cSDE to PTH diagnosis (days), mean (SD)	52.0 (31.7)
Time from cSDE to PTH diagnosis (days), range	23 ~ 152
PTH diagnosed before cranioplasty, *n* (%)	8 (38.1%)
PTH diagnosed after cranioplasty, *n* (%)	13 (61.9%)

Among symptomatic patients, presenting features included decreased level of consciousness in 12 patients (75.0%), bulging or tense cranial defect in 9 patients (56.3%), headache in 1 patient (6.3%), and new or worsening focal deficits in 2 patients (12.5%).

The mean interval from cSDE diagnosis to PTH diagnosis was 52 days (range: 23–152 days). Notably, 13 patients (61.9%) developed PTH after cranioplasty.

### 3.2 Radiological features

Initially, cSDE appeared as a hypodense crescentic collection over the contralateral convexity, often with varying degrees of midline shift. As time progressed, ventricular enlargement became apparent, with Evans’ index increasing from 0.26 ± 0.04 at cSDE diagnosis to 0.35 ± 0.05 at PTH diagnosis (*p* < 0.001).

The mean maximal thickness of cSDE was 8.7 ± 2.3 mm. At cSDE diagnosis, 12 patients (57.1%) had midline shift >5 mm. The progression of Evans’ index is detailed in Table 3.

**Table 3:** Radiological parameters.

Parameter	At cSDE diagnosis	At PTH diagnosis	*p*-value
Evans’ index, mean ± SD	0.26 ± 0.04	0.35 ± 0.05	<0.001
cSDE thickness (mm), mean ± SD	8.7 ± 2.3	5.2 ± 2.1	<0.001
Midline shift (mm), mean ± SD	7.8 ± 2.9	3.1 ± 1.8	<0.001

### 3.3 Treatment and outcomes

The treatment modalities and outcomes are summarized in Table 4.

**Table 4:** Treatment modalities and outcomes.

Treatment modality	Number of patients (*N* = 21)	Percentage	Outcome details
Conservative management	5	23.8%	• 4 (80.0%) eventually required surgical intervention due to progression • 1 (20.0%) showed spontaneous resolution
Burr-hole drainage/Ommaya reservoir	12	57.1%	• Temporary improvement in all 12 patients (100%) • Recurrence in 7 patients (58.3%) • Ommaya reservoir placement in 2 patients
Cranioplasty alone	6	28.6%	• Complete cSDE resolution: 4 patients (66.7%) • Partial cSDE resolution: 2 patients (33.3%) • Subsequent hydrocephalus requiring VP shunt: 4 patients (66.7%)
VP shunt alone	2	9.5%	• Both patients (100%) subsequently required cranioplasty
Staged cranioplasty followed by VP shunt	7	33.3%	• Cranioplasty performed first • VP shunt placed when hydrocephalus became evident postoperatively
Simultaneous cranioplasty and VP shunt	4	19.0%	• Performed in patients with concurrent symptomatic PTH and cSDE
Subdural-peritoneal shunt	2	9.5%	• For refractory subdural effusion • One patient underwent VP shunt + SPS shunt followed by elective cranioplasty

**Figure 2:**
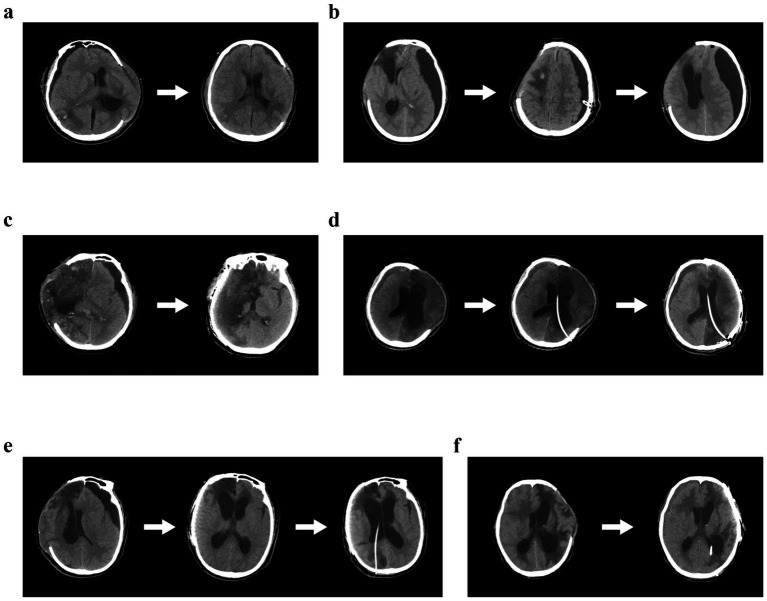
Radiographic progression of cSDE and PTH following treatment. **(a)** Following conservative treatment, both the cSDE and PTH showed resolution. **(b)** After burr hole drainage of the cSDE, the effusion improved but it may recur without continuous drainage. **(c)** After cranioplasty, the cSDE resolved, and no PTH developed. **(d)** VP shunt placement was initially performed to address the hydrocephalus, followed by cranioplasty. **(e)** Following cranioplasty, the cSDE resolved, but the PTH subsequently worsened, requiring VP shunt placement. **(f)** After conservative treatment, the cSDE resolved, and the patient simultaneously underwent VP shunt placement and cranioplasty.

*Conservative management*: Five patients (23.8%) were initially managed conservatively with observation, head-down positioning, and fluid management. Of these, four (80.0%) eventually required surgical intervention due to progression, while one (20.0%) showed spontaneous resolution, as shown in Figure 2a.

*Burr-hole drainage/ommaya reservoir*: Twelve patients (57.1%) underwent burr-hole drainage, with two receiving Ommaya reservoir placement. Temporary improvement was observed in all 12 patients (100%), but recurrence occurred in seven (58.3%), as shown in Figure 2b.

*Cranioplasty alone*: Six patients (28.6%) underwent cranioplasty as the primary intervention for chronic subdural effusion. Following cranioplasty, the effusion resolved completely in four patients (66.7%), as shown in Figure 2c, and partially in two (33.3%). However, four of these patients (66.7%) subsequently developed symptomatic hydrocephalus requiring a ventriculoperitoneal shunt.

*VP shunt performed first*: Two patients (9.5%) with established hydrocephalus at presentation underwent VP shunt placement without prior cranioplasty. Both of these patients subsequently required cranioplasty, as shown in Figure 2d.

*Cranioplasty followed by elective VP shunt*: Seven patients (33.3%) underwent staged management, with cranioplasty performed first, followed by VP shunt once hydrocephalus became evident postoperatively, as shown in Figure 2e.

*Simultaneous cranioplasty and VP shunt*: Four patients (19.0%) with concurrent symptomatic post-traumatic hydrocephalus and chronic subdural effusion underwent simultaneous procedures, as shown in Figure 2f.

At 6-month follow-up, overall outcomes demonstrated that 14 patients (66.7%) achieved a favorable outcome (GOS 4–5), while seven patients (33.3%) had an unfavorable outcome (GOS 1–3), including two patients (9.5%) who died. Regarding complications, shunt infection occurred in one patient (4.8%), shunt malfunction requiring revision in two (9.5%), and postoperative hemorrhage in one patient (4.8%).

### 3.4 Factors associated with outcome

Univariate and multivariate logistic regression analyses were performed to identify factors associated with unfavorable outcome (GOS 1–3) at 6 months (Table 5). In univariate analysis, age >50 years, admission GCS ≤ 8, midline shift >10 mm at cSDE diagnosis, Evans’ index >0.35 at PTH diagnosis, and the need for subsequent surgery were significantly associated with poor outcome. Time to cSDE diagnosis >14 days did not show a statistically significant association. In multivariate analysis, admission GCS ≤ 8 (OR = 3.12; 95% CI: 1.17–8.32; *p* = 0.023) and midline shift >10 mm at cSDE diagnosis (OR = 3.67; 95% CI: 1.42–9.48; *p* = 0.007) remained independent predictors of unfavorable outcome. The need for subsequent surgery approached but did not reach statistical significance (OR = 2.89; 95% CI: 0.96–8.67; *p* = 0.058). Age >50 years, Evans’ index >0.35, and time to cSDE diagnosis >14 days were not included in the multivariate model due to lack of significance or stepwise regression exclusion.

**Table 5:** Logistic regression analysis of factors associated with unfavorable outcome.

Variable	Univariate OR (95% CI)	*p*-value	Multivariate OR (95% CI)	*p*-value
Age > 50 years	2.54 (1.12–5.78)	0.026	Not analyzed	Not analyzed
Admission GCS ≤ 8	3.76 (1.48–9.55)	0.005	3.12 (1.17–8.32)	0.023
Time to cSDE diagnosis > 14 days	1.89 (0.84–4.26)	0.123	Not analyzed	Not analyzed
Midline shift > 10 mm at cSDE diagnosis	4.28 (1.73–10.59)	0.002	3.67 (1.42–9.48)	0.007
Evans’ index > 0.35 at PTH diagnosis	2.95 (1.21–7.19)	0.017	Not analyzed	Not analyzed
Need for subsequent surgery	5.43 (2.08–14.18)	<0.001	2.89 (0.96–8.67)	0.058

## 4 Discussion

### 4.1 Summary of Main findings

This single-center retrospective study analyzed the clinical characteristics, progression patterns, and treatment outcomes of patients with concomitant PTH and cSDE following DC for severe TBI. Our main findings can be summarized as follows:

First, the mean interval from DC to cSDE diagnosis was 15.6 days, which is similar to the 13-day average reported by Su et al. (2) and Wang et al. (5). This finding supports the recommendation for routine CT surveillance at 2–3 weeks post-DC regardless of clinical status (2, 3).

Second, a substantial proportion of patients (23.8%) were asymptomatic at cSDE diagnosis, underscoring the importance of routine imaging for early detection (2, 4). However, among symptomatic patients, decreased consciousness and bulging cranial defects were the most common presentations, similar to findings from Yang et al. (16).

Third, and most importantly, 66.7% of patients who underwent cranioplasty for cSDE subsequently developed symptomatic hydrocephalus requiring VP shunt placement. This phenomenon—cranioplasty “unmasking” underlying hydrocephalus—has been increasingly recognized in the literature (4, 10, 11, 13). Wang et al. (4) described a patient whose cSDE resolved after cranioplasty but who then developed hydrocephalus requiring shunt, while Salunke et al. (11) reported that 3 of 7 patients (43%) required shunt after cranioplasty. Our findings corroborate these observations and suggest that this sequence may be more common than previously appreciated.

Fourth, the rate of subsequent hydrocephalus in our cSDE cohort was relatively high (71.4%), which is higher than the 46.2% rate reported by Su et al. (2). This further reinforces the concept that cSDE and PTH are pathophysiologically linked and may represent different manifestations of the same underlying CSF dynamics disturbance.

### 4.2 Pathophysiological mechanisms

The concurrent development of PTH and cSDE after DC can be explained by several complementary mechanisms.

The “arachnoid valve” theory: Traumatic tearing of the arachnoid membrane creates a one-way valve allowing CSF to flow into the subdural space (4, 5, 11). This mechanism is supported by intraoperative observations and the fact that cSDE rarely occurs after DC in non-traumatic conditions such as stroke (12, 16).

The “pressure gradient” theory: Following DC, the brain is exposed to atmospheric pressure, causing outward herniation through the cranial defect. This creates a pressure differential between the two hemispheres, enlarging the contralateral subdural space and promoting fluid accumulation (4, 5, 12). The rapid brain shift during surgery may also tear contralateral bridging veins, contributing to fluid collection (6, 17). This mechanism is clinically supported by the risk factor analysis of Yuan et al. (18), who identified that in patients undergoing unilateral decompressive craniectomy, the presence of temporal hematoma or contusion on the non-operative side was an independent risk factor for ipsilateral subdural effusion, while frontal hematoma or contusion on the non-operative side predicted contralateral effusion. These findings suggest that focal brain injury patterns on the side opposite to the craniectomy can exacerbate the pressure gradient and predispose to localized fluid accumulation.

In this context, the pathophysiology of cSDE can also be framed within the concept of the “syndrome of the trephined” (SoT). SoT, also known as “sinking skin flap syndrome,” is an under-recognized complication following DC, characterized by a spectrum of delayed neurological and physical symptoms that improve after cranioplasty (19). A key mechanism of SoT involves the exposure of the decompressed hemisphere to atmospheric pressure. This external pressure alters the transmural pressure gradient at the level of the superior sagittal sinus and the arachnoid granulations (granulations of Pacchioni), thereby impairing the normal pressure-dependent CSF resorption process (19). Consequently, this disturbance in CSF dynamics can lead to both the accumulation of fluid in the subdural space (cSDE) and ventricular enlargement (hydrocephalus), either in isolation or concurrently. This perspective reinforces that cSDE and PTH are not merely separate complications but rather different manifestations of a shared, underlying derangement in CSF homeostasis triggered by the cranial defect.

The “CSF absorption” theory: Arachnoid granulations function as pressure-dependent one-way valves (4, 8). After DC, the normal dicrotic ICP waveform is flattened, reducing the pulsatile drive for CSF absorption and leading to fluid accumulation in both the subdural space and ventricles (1, 4, 10). This mechanism explains why both cSDE and hydrocephalus can coexist—they represent different compartments for CSF accumulation when the primary absorptive pathway is impaired.

The “cranioplasty unmasks hydrocephalus” phenomenon: When the cranial defect is present, the brain can expand outward, partially decompressing the ventricular system. This may mask underlying hydrocephalus even when CSF absorption is impaired. Cranioplasty restores the “closed box,” eliminating this compensatory mechanism and allowing ventricular enlargement to become apparent (4, 11, 13). This explains why some patients appear to deteriorate after successful cSDE treatment—the underlying hydrocephalus was present all along but clinically silent.

### 4.3 Discussion of a typical case

A 65-year-old male patient presented to the emergency department of a local hospital with a head injury caused by a fall. A head CT scan diagnosed an acute right frontotemporoparietal subdural hematoma and multiple cerebral contusions. He underwent decompressive craniectomy on the day of injury and remained comatose postoperatively. On postoperative day 5, a follow-up head CT revealed a subdural effusion contralateral to the craniectomy site, which progressively increased in volume over subsequent days. The patient was then transferred to our hospital for further evaluation and management.

**Figure 3:**
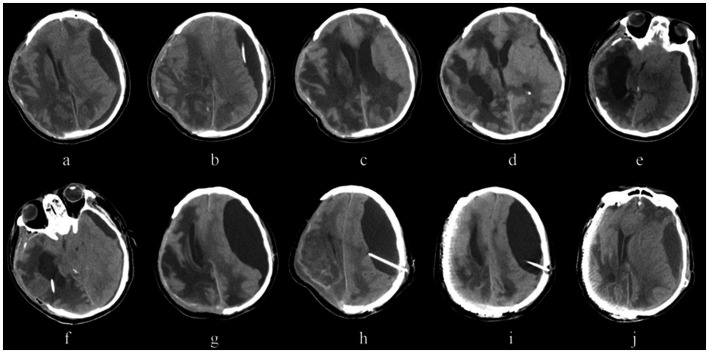
Serial CT images from a representative case. **(a)** Initial head CT scan on admission shows status post‑right frontotemporoparietal decompressive craniectomy, with a subdural effusion over the left frontotemporoparieto‑occipital region causing a 13 mm midline shift to the right. **(b)** Head CT after left parietal burr‑hole drainage of the subdural effusion. **(c)** Follow‑up CT showing gradual reduction of the subdural effusion and evidence of bilateral ventricular dilation. **(d)** CT performed one week after removal of the subdural drain reveals recurrence of the subdural effusion and significant enlargement of the right temporal horn of the lateral ventricle. **(e)** Repeat CT three days later shows persistent left frontotemporal subdural effusion and further enlargement of the right temporal horn. **(f)** CT following placement of a right temporal horn ventriculoperitoneal shunt. **(g)** Over the subsequent two weeks, CT demonstrates progressive enlargement of the left frontotemporoparietal subdural effusion, with no improvement after ventriculoperitoneal shunt valve pressure adjustments. **(h)** CT after placement of a subdural‑peritoneal shunt. **(i)** CT obtained one month later, after the patient’s condition had stabilized and cranioplasty was performed. **(j)** CT one month post‑cranioplasty shows gradual decrease of the subdural effusion; both hydrocephalus and subdural effusion are well‑controlled.

Upon admission, a head CT scan (Figure 3a) showed the post-right frontotemporoparietal decompressive craniectomy status, with a subdural effusion over the left frontotemporoparieto-occipital region causing a 13 mm midline shift. The patient subsequently underwent left parietal burr-hole drainage of the subdural effusion (Figure 3b). Postoperatively, the patient remained comatose, but follow-up CT scans demonstrated gradual reduction of the subdural effusion and evidence of bilateral ventricular dilation (Figure 3c). The subdural drain was removed 3 days later.

One week after drain removal, a CT scan revealed recurrence of the subdural effusion and significant enlargement of the right temporal horn of the lateral ventricle (Figure 3d). A repeat CT 3 days later showed persistent left frontotemporal subdural effusion and further enlargement of the right temporal horn (Figure 3e).

A right temporal horn-ventriculoperitoneal shunt was placed (Figure 3f). However, over the subsequent 2 weeks, the left frontotemporoparietal subdural effusion progressively enlarged, and adjustments to the ventriculoperitoneal shunt valve pressure failed to improve the effusion (Figure 3g). Consequently, a subdural-peritoneal shunt was placed (Figure 3h).

One month later, after the patient’s condition stabilized, a cranioplasty was performed (Figure 3i). One month post-cranioplasty, the subdural effusion gradually decreased, and both the hydrocephalus and subdural effusion were well-controlled (Figure 3j).

This case was notably intricate, involving a succession of surgical interventions. Post-discharge follow-up revealed that the patient remains in a state of confusion and is currently undergoing further rehabilitative therapy at a specialized rehabilitation facility. This case underscores the multifaceted and dynamic nature of managing patients with concurrent post-traumatic hydrocephalus and subdural effusion. It highlights that the therapeutic approach and surgical strategy cannot be rigidly protocol-driven; instead, they must be continuously adapted based on the patient’s evolving clinical and radiological status to achieve optimal disease control and outcomes. The complexity of this case serves as a potent reminder of the need for individualized, stepwise management and vigilant long-term follow-up in this challenging patient population.

### 4.4 Comparison with previous studies

Our findings are consistent with and extend previous reports on this topic.

*Incidence and timing*: The incidence of cSDE in our series (4.8%) falls within the reported range of 4.4%–7.3% (2–5). The mean time to cSDE diagnosis (15.6 days) closely matches the 13 days reported by Su et al. (2) and the 2–3 week window suggested by multiple authors (3, 16).

*Treatment outcomes*: Conservative management was effective in only 9.5% of our patients, which is similar to the experience of Su et al. (2). Although burr-hole drainage provided temporary improvement, the recurrence rate was high (58.3%), mirroring the experience of Salunke et al. (11) and Zhu et al. (9).

*Cranioplasty efficacy*: The complete resolution rate of cSDE after cranioplasty in our series was 66.7%, which is lower than the results reported by Wan et al. (3) and Salunke et al. (11). However, 66.7% of our cranioplasty patients subsequently developed hydrocephalus, a phenomenon that highlights an important caveat not emphasized in all previous reports.

*Hydrocephalus rates*: The incidence of PTH in our cSDE patients (81.0%) was considerably higher than the 46.2% reported by Su et al. (2), but is consistent with the recognition that cSDE patients represent a high-risk group for eventual shunt dependency (1, 4).

### 4.5 Clinical implications and proposed algorithm

**Figure 4:**
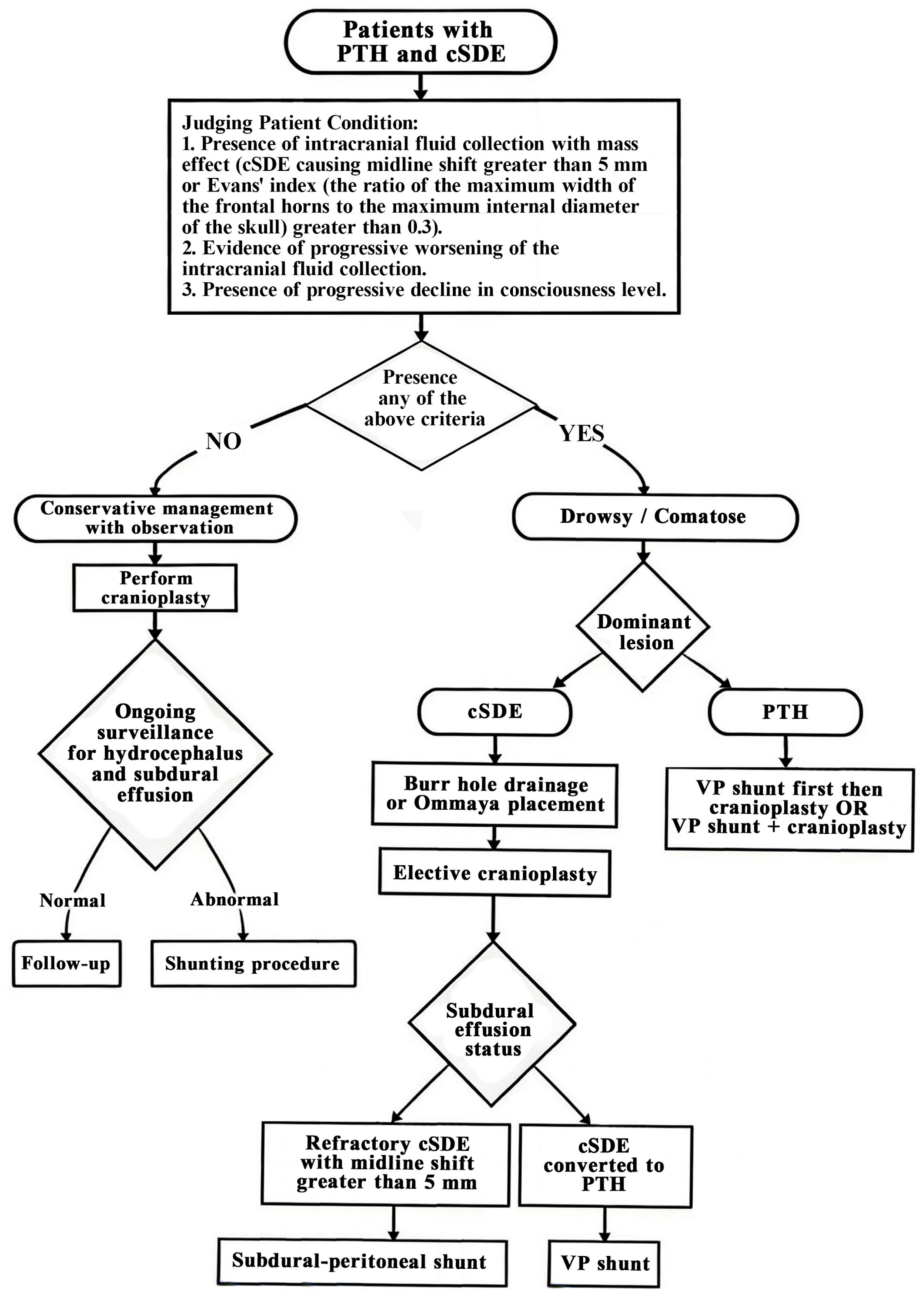
Management algorithm for PTH patients with concomitant cSDE.

Based on our findings and synthesis of the literature, we propose the following management algorithm for patients with PTH and concomitant cSDE (Figure 3). The algorithm begins with diagnosis and classification, requiring radiological confirmation of both conditions (cSDE on the contralateral convexity and Evans’ index >0.3), assessment of clinical symptoms attributable to each condition, and determination of which condition is dominant both clinically and radiologically Figure 4.

For patients with symptomatic cSDE as the dominant problem and no evidence of significant hydrocephalus (Evans’ index <0.35, no clinical symptoms of hydrocephalus), we recommend considering cranioplasty first, followed by close clinical and radiological surveillance post-cranioplasty, with preparation for possible shunt placement if hydrocephalus emerges. For patients with symptomatic hydrocephalus as the dominant problem and asymptomatic or minimal cSDE, VP shunt placement should be performed first, followed by elective cranioplasty, with monitoring for resolution of cSDE post-shunt.

For patients with both conditions symptomatic, clinicians may consider either simultaneous cranioplasty and VP shunt or a staged approach with short interval between procedures. For patients with refractory cSDE after prior treatments, options include Ommaya reservoir placement with serial aspirations (9), subduroperitoneal shunting if other measures fail (2, 6), or consideration of temporal muscle sticking as described by Wan et al. (3).

Surveillance recommendations include routine CT at 2–3 weeks post-DC regardless of symptoms (2, 3), monthly clinical evaluation and CT until 3 months post-cranioplasty, and immediate imaging for any neurological deterioration.

### 4.6 Prognostic implications

Our multivariate analysis identified admission GCS ≤ 8 and midline shift >10 mm at cSDE diagnosis as independent predictors of unfavorable outcome at 6 months. These findings suggest that initial injury severity and the degree of mass effect from cSDE are the most robust independent predictors of long-term prognosis in this complex patient population. Clinically, this implies that patients with these risk factors warrant more aggressive surveillance and earlier intervention.

### 4.7 Limitations

This study has several limitations that should be acknowledged, including the retrospective design which introduces potential selection bias and limits our ability to establish causal relationships, as treatment decisions were made at the discretion of individual neurosurgeons rather than according to a standardized protocol; the relatively small sample size (21 patients), which limits statistical power and precludes subgroup analyses, although given the rarity of this combined condition, even small series provide valuable clinical insights; the single-center nature of the study, which may limit generalizability to other populations or healthcare settings with different practice patterns; the lack of a control group (e.g., PTH without cSDE), which limits our ability to determine whether the outcomes and complication rates are specific to the combined condition or simply reflect the natural history of PTH; the follow-up period of 6 months, which may be insufficient to capture late complications or delayed hydrocephalus development, as longer follow-up might reveal higher rates of eventual shunt dependency; the absence of routine CSF dynamics studies (e.g., infusion tests, ICP monitoring), which might provide more objective evidence of hydrocephalus and guide treatment decisions; and finally, the lack of standardized criteria for when to intervene surgically, which introduces variability in treatment thresholds across different surgeons.

### 4.8 Future directions

Future research endeavors should address the aforementioned limitations through prospective multicenter collaborations with larger, more diverse patient cohorts to enhance statistical power and generalizability. The implementation of standardized treatment protocols and uniform outcome measures is essential to enable meaningful comparisons across studies and institutions. Extended follow-up periods are warranted to comprehensively assess late-onset complications and the potential for delayed hydrocephalus development requiring shunting. Furthermore, incorporation of advanced cerebrospinal fluid dynamics studies, including infusion testing and intracranial pressure monitoring, could provide crucial insights into the underlying pathophysiology and guide more targeted therapeutic interventions. Well-designed randomized controlled trials comparing different treatment sequences (e.g., cranioplasty-first versus shunt-first approaches) are urgently needed to establish evidence-based management algorithms. Finally, the development and validation of robust predictive models incorporating clinical, radiological, and potentially biochemical parameters would facilitate early identification of patients at highest risk for post-cranioplasty hydrocephalus, enabling proactive surveillance and timely intervention. Such studies will collectively contribute to optimizing the management of this challenging clinical entity and improving patient outcomes.

## 5 Conclusion

Post-traumatic hydrocephalus accompanied by contralateral subdural effusion represents a distinct and clinically challenging entity following decompressive craniectomy for severe traumatic brain injury. Our single-center retrospective study demonstrates that these conditions are pathophysiologically linked, often follow a predictable temporal progression, and require individualized management strategies. This conclusion is in keeping with the recent consensus-based recommendations for the diagnosis and surgical management of cranioplasty and post-traumatic hydrocephalus from a European panel, which also emphasize a staged and individualized approach (20).

The key conclusions drawn from this study underscore the critical importance of routine CT surveillance at 2 to 3 weeks post-DC for the early detection of cSDE, as a substantial proportion of patients remain asymptomatic during the initial phase. Cranioplasty proves to be an effective therapeutic option for symptomatic cSDE, achieving high rates of effusion resolution. Nevertheless, clinicians must remain vigilant that cranioplasty may unmask underlying hydrocephalus in a significant subset of patients, necessitating close postoperative monitoring and timely ventriculoperitoneal shunt placement. The notably high rate of subsequent hydrocephalus observed in our cSDE cohort reinforces the notion that these patients constitute a high-risk group warranting enhanced and prolonged surveillance.

Given the complexity of this combined condition, treatment strategies should be carefully individualized based on the clinically dominant pathology. Available options range from conservative management and burr-hole drainage to cranioplasty alone, VP shunt alone, or various combinations of staged and simultaneous procedures. To guide clinical decision-making, we propose a practical treatment algorithm that emphasizes the necessity of close follow-up. By recognizing and proactively addressing the unique challenges posed by this dual pathology, neurosurgeons can optimize outcomes and minimize complications in this complex patient population.

Looking ahead, further prospective studies with larger, multi-institutional cohorts and standardized protocols are essential to validate our findings and refine management strategies. The growing recognition of this clinical entity, as reflected in the expanding body of literature, highlights its significance in the post-DC care of TBI patients and underscores the imperative for continued research in this area.

## Data Availability

The original contributions presented in the study are included in the article/supplementary material, further inquiries can be directed to the corresponding author.
